# *Bmp6* Expression Can Be Regulated Independently of Liver Iron in Mice

**DOI:** 10.1371/journal.pone.0084906

**Published:** 2014-01-13

**Authors:** Zhuzhen Zhang, Xin Guo, Carolina Herrera, Yunlong Tao, Qian Wu, Aimin Wu, Hao Wang, Thomas B. Bartnikas, Fudi Wang

**Affiliations:** 1 Laboratory of Nutrition and Metabolism, Center for Nutrition and Health, Department of Nutrition, Institute of Nutrition and Food Safety, School of Public Health, Collaborative Innovation Center for Diagnosis and Treatment of Infectious Diseases, Zhejiang University, Hangzhou, Zhejiang, China; 2 Key Laboratory of Nutrition and Metabolism, Institute for Nutritional Sciences, Shanghai Institutes for Biological Sciences, Chinese Academy of Sciences, Graduate School of the Chinese Academy of Sciences, Shanghai, China; 3 Department of Pathology and Laboratory Medicine, Brown University, Providence, Rhode Island, United States of America; National Institute of Child Health and Human Development, United States of America

## Abstract

The liver is the primary organ for storing iron and plays a central role in the regulation of body iron levels by secretion of the hormone Hamp1. Although many factors modulate *Hamp1* expression, their regulatory mechanisms are poorly understood. Here, we used conditional knockout mice for the iron exporter *ferroportin1* (*Fpn1*) to modulate tissue iron in specific tissues in combination with iron-deficient or iron-rich diets and transferrin (Tf) supplementation to investigate the mechanisms underlying *Hamp1* expression. Despite liver iron overload, expression of bone morphogenetic protein 6 (*Bmp6*), a potent-stimulator of *Hamp1* expression that is expressed under iron-loaded conditions, was decreased. We hypothesized that factors other than liver iron must play a role in controlling *Bmp6* expression. Our results show that erythropoietin and Tf-bound iron do not underlie the down-regulation of *Bmp6* in our mice models. Moreover, *Bmp6* was down-regulated under conditions of high iron demand, irrespective of the presence of anemia. We therefore inferred that the signals were driven by high iron demand. Furthermore, we also confirmed previous suggestions that Tf-bound iron regulates *Hamp1* expression via Smad1/5/8 phosphorylation without affecting *Bmp6* expression, and the effect of Tf-bound iron on *Hamp1* regulation appeared before a significant change in *Bmp6* expression. Together, these results are consistent with novel mechanisms for regulating *Bmp6* and *Hamp1* expression.

## Introduction

Iron metabolism is a complex yet highly coordinated process. Hamp1, a short peptide secreted primarily by the liver [1], is vitally important for maintaining iron homeostasis throughout the body. Hamp1 binds to the non-heme iron exporter ferroportin 1 (Fpn1) and induces its internalization and degradation; Fpn1 is the only known non-heme iron exporter [2]–[3]. Many factors regulate *Hamp1* expression, including iron overload [4] and iron deficiency, which induce and inhibit, respectively, the expression of this protein. *Hamp1* expression is also regulated by inflammatory cytokines and erythropoietic factors [5]. However, the mechanisms that regulate *Hamp1* expression remain poorly understood.

Bone morphogenetic proteins (BMPs) play an essential role in *Hamp1* regulation [6]–[7]. The binding of BMPs to BMP receptors and the co-receptor Hemojuvelin (Hjv) triggers the phosphorylation of the transcription factors Smad1/5/8. Phosphorylated Smad1/5/8 (p-Smad1/5/8) then forms a complex with Smad4 and translocates to the nucleus, where it binds the *Hamp1* promoter and modulates *Hamp1* expression [8]. Bmp6 has been shown to be a key regulator of *Hamp1* expression *in vivo* [9], and *Bmp6* expression is usually correlated with liver iron content [10]–[11]. Mice with *Bmp6* deficiency[12], *Hjv* deficiency [13]–[14], or hepatocyte-specific *Hjv* [15] or *Smad4* [16] deletion all have severely reduced *Hamp1* expression and a phenotype that closely resembles *Hamp1*-deficient mice [1]; therefore, the Bmp6/Hjv/p-Smad1/5/8 pathway clearly plays a key role in *Hamp1* regulation [17].

Another critical regulator of *Hamp1* expression is transferrin (Tf)-bound iron, the primary source of iron for erythroid cells [18]. The levels of Tf-bound iron generally reflect the body's balance between iron supply and iron demand [19]. Both patients and mice with mutations in the *Tf* gene develop a condition called hypotransferrinemia, in which the body produces little or no Tf-bound iron and develops severe anemia [20]–[21]. Under these conditions, *Hamp1* levels are reduced considerably. Moreover, Tf supplementation restores *Hamp1* expression, demonstrating the essential role that Tf-bound iron plays in *Hamp1* regulation [22]. In addition, patients and mice with hypotransferrinemia also exhibit massive erythropoietic drive and hypoxia, both of which are factors that inhibit *Hamp1* regulation [5]. Studies have shown that Tf-bound iron regulates *Hamp1* expression through the phosphorylation of Smad1/5/8 (p-Smad1/5/8) [23]. This regulation is mediated by an interaction between Tf-bound iron and transferrin receptor 1 (Tfr1) or 2 (Tfr2), with the membrane-bound protein Hfe acting as an intermediate factor [24]. Mutations in *Hfe* [25] or *Tfr2* [26] lead to *Hamp1* down-regulation, and the combined deletion of both *Hfe* and *Tfr2* results in extremely severe *Hamp1* down-regulation, with decreased phosphorylation of Smad1/5/8, Erk1 and Erk2 [27]. Together, these results suggest that Tf-bound iron can modulate *Hamp1* expression through the Hfe/Tfr complex, with p-Smad1/5/8 and p-Erk1/2 serving as intermediate signaling molecules.

In this study, we dissected the factors involved in *Hamp1* regulation and found that factors other than liver iron levels can regulate the expression of *Bmp6*. Our data suggest that the signal for *Bmp6* down-regulation may arise from the driving force of high iron demand. Our study also confirms previous reports that Tf-bound iron regulates *Hamp1* expression via p-Smad1/5/8 without affecting *Bmp6* expression.

## Materials and Methods

### Ethics statement

All mice were handled in accordance with our institution's humane animal care policies. The experimental protocols were approved by the Institutional Animal Care and Use Committee of The Institute for Nutritional Sciences, Shanghai Institutes for Biologic Sciences, and Chinese Academy of Sciences.

### Animals and animal treatment

The generation of *Fpn1^Alb/Alb^* and *Fpn1^Alb/Alb;LysM/LysM^* mice was described previously [28]. *Tek-Cre* mice [29], which express Cre recombinase under the *Tek* receptor tyrosine kinase promoter/enhancer, were purchased from the Jackson Laboratory and maintained on a 129/SvEvTac background, then crossed with *Fpn1^flox/flox^* mice to generate *Fpn1^Tek/Tek^* mice. *Fpn1^flox/flox^* mice were also maintained on a 129/SvEvTac background. *Fpn1* was systemically deleted in embryonic *Fpn1^Tek/Tek^* mice due to maternal Cre recombinase expression [29]–[30]. The mice were fed a normal rodent laboratory diet (containing 232 mg iron/kg) obtained from SLRC Laboratory Animal Co., Ltd. (Shanghai, China) unless otherwise specified. Age-matched male mice were used in separate experiments. To induce various serum levels of Tf-bound iron, 2-month-old *Fpn1^flox/flox^* and *Fpn1^Alb/Alb;LysM/LysM^* mice were fed an iron-deficient diet for 0, 2, 4 or 8 days. The mice then received an intraperitoneal injection of 10 mg human holo-Tf (or an equal volume of PBS), then given *ad libitum* access to normal rodent diet overnight to ensure high saturation of the supplemented Tf. For experiments requiring iron loading followed by the mobilization of iron from internal stores, 3-week-old *Fpn1^flox/flox^* and *Fpn1^Alb/Alb^* mice were fed an iron-rich diet for one week, and then placed on an iron-deficient diet for one month. Standard (50 mg iron/kg), iron-deficient (0.9 mg iron/kg), and iron-rich (8.3 g of carbonyl iron/kg) diets were egg white-based AIN-76A diets (Research Diets, Inc., New Brunswick, NJ).

### Measurement of serum iron, hematological parameters, tissue non-heme iron,and tissue iron staining

Assays to measure serum iron levels, hematological parameters, quantitative measurements of tissue non-heme iron, and Perls' Prussian Blue and DBA iron staining were performed as previously described [31].

### Western blot analysis and qRT-PCR

Western blot analyses and qRT-PCR were performed as previously described [31]. All primary antibodies were purchased from Cell Signaling Technology (Danvers, MA). Western blots were analyzed with densitometry using Bio-Rad Quantity One software. The qRT-PCR data were normalized to the internal control (*β-actin*) and are presented as the relative expression level (calculated using the 2^ΔΔ^Ct method. The primers used for the qRT-PCR experiments are listed in Table S1 in File S1.

### Statistical analysis

Summary data are presented as the mean ± standard deviation (SD). The Student's *t*-test was used to compare the groups, and differences with P<0.05 are considered to be statistically significant.

## Results

### *Bmp6* and *Hamp1* expression are decreased in iron-loaded *Fpn1^Tek/Tek^* mice with severe iron-deficiency anemia

**Figure 1:**
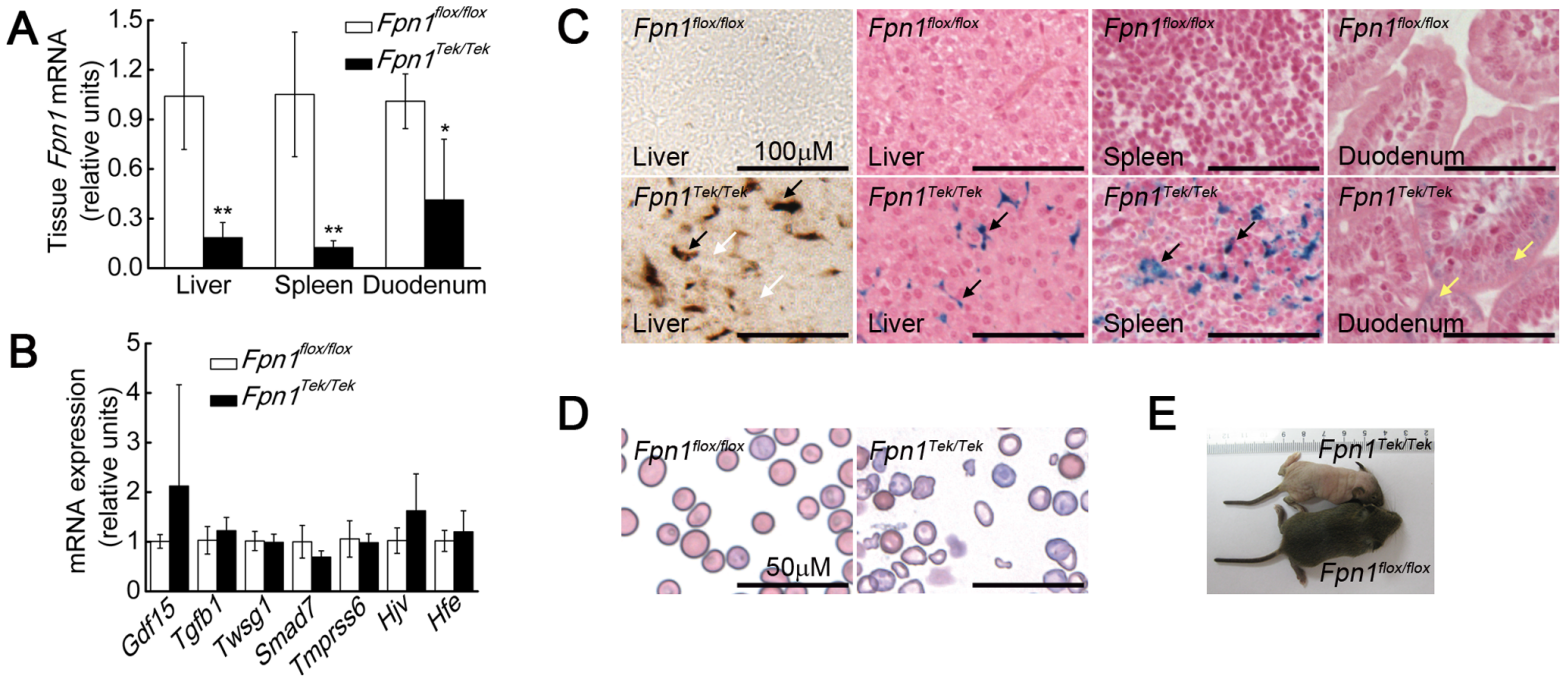
*Fpn1^Tek/Tek^* mice develop anemia and have iron loading in their hepatocytes, macrophages, and duodenal enterocytes. (A) *Fpn1* mRNA levels in the liver, spleen and duodenum. (B) Liver mRNA levels of genes implicated in *Hamp1* regulation (n = 6–7 per group). (C) DAB iron staining in the liver (left panels; brown staining indicates iron), Perls' Prussian Blue iron staining in the liver, spleen, and duodenum (right panels; brown staining indicates iron). Iron accumulated in hepatocytes (White arrowheads), liver kupffer cells and spleen macrophages (Black arrowheads), and duodenum enterocytes (Yellow arrowheads). (D) Wright-Giemsa-stained peripheral blood smears. (E) Photographs of 13–15-day-old male *Fpn1^flox/flox^* and *Fpn1^Tek/Tek^* mice. DAB iron staining was used to detect tissue iron levels when Prussian Blue staining was not sufficiently sensitive. Images were captured on an Olympus BX61 microscope with a UPlanApo 20×/0.70 or 40×/0.85 objective, Q Imaging QICAM camera and Q Capture 2.90.1 Quantitative Imaging software. Summary data are presented as mean ± SD. *P<0.05; **P<0.01.

**Figure 2:**
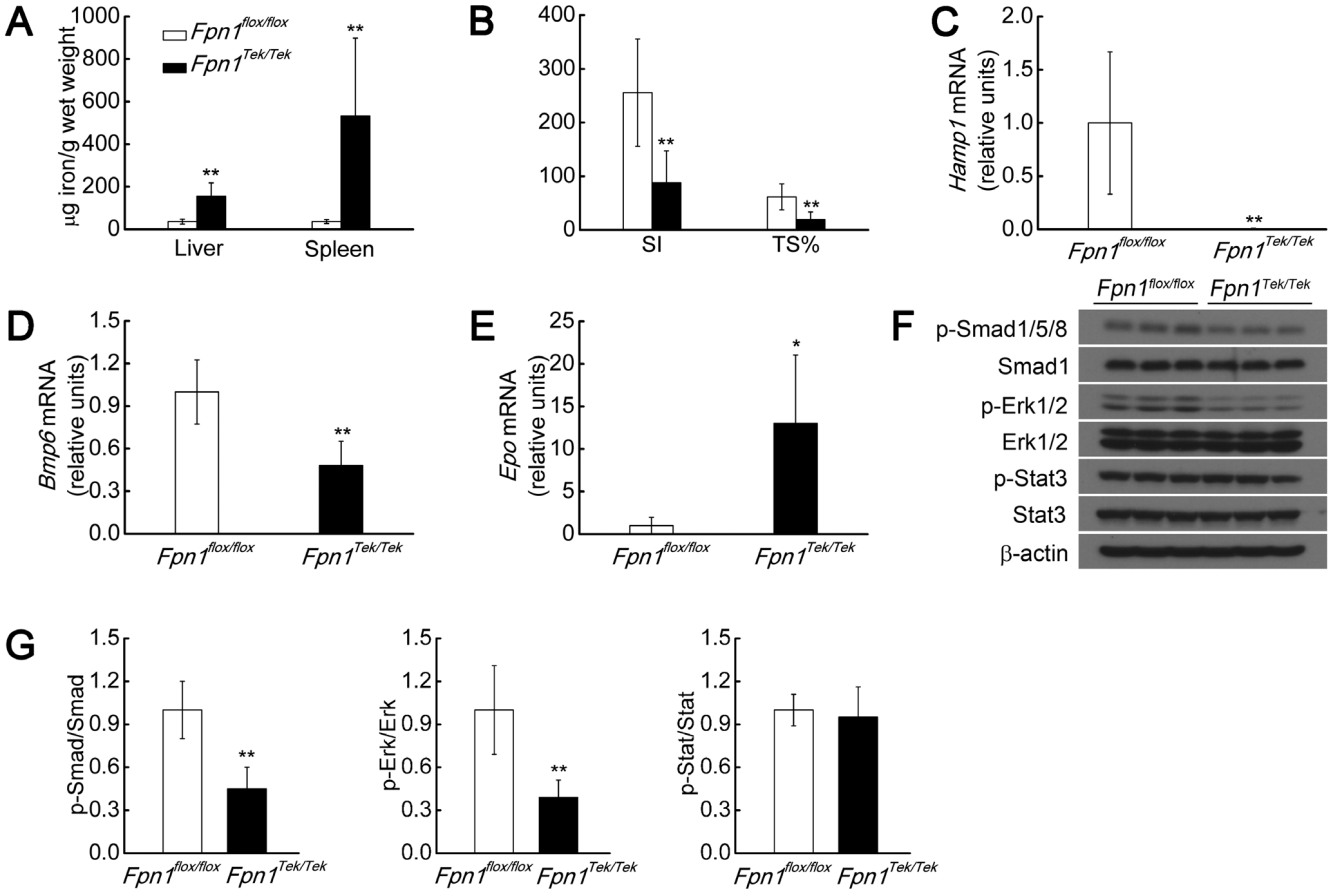
*Fpn1^Tek/Tek^* mice have liver iron loading but decreased *Bmp6* and *Hamp1* expression. (A) Liver and spleen non-heme iron concentrations. (B) Serum iron concentration (SI, µg/dL) and percent Tf saturation (TS%). (C–E) Liver mRNA levels of *Hamp1* (C), *Bmp6* (D) and *Epo* (E). (F) Liver p-Smad1/5/8, Smad1, p-Erk1/2, Erk1/2, p-Stat3, Stat3 and β-actin protein levels were measured in 13–15-day-old male *Fpn1^flox/flox^* and *Fpn1^Tek/Tek^* mice (n = 6–7 per group), (G) Summary of the results in (F), quantitated using densitometry. Summary data are presented as mean ± SD. *P<0.05; **P<0.01.

The mechanisms that regulate *Hamp1* expression are not fully understood. Therefore, we used mouse models with a conditional knockout in the non-heme iron exporter *Fpn1* to establish stable iron levels in specific tissues. We first established a baseline phenotype of *Fpn1* deficiency to which we could compare the results of subsequent experiments. Because global *Fpn1* deletion is embryonic lethal, we used *Fpn1^Tek/Tek^* mice [29]–[30], in which the expression of Cre recombinase is driven by the receptor tyrosine kinase *Tek* promoter/enhancer, resulting in the deletion of *Fpn1* in the maternal germline and in endothelial cells, without causing embryonic lethality. Thirteen-to-fifteen-day-old *Fpn1^Tek/Tek^* mice had decreased *Fpn1* mRNA levels in the liver, spleen, and duodenum; moreover, the expression of other genes implicated in iron metabolism was unchanged (Figure 1A, 1B). Total iron levels were increased in the liver and spleen of the *Fpn1^Tek/Tek^* mice, and iron accumulation was detected primarily in macrophages and duodenal enterocytes. The iron levels in the hepatocyte were also increased, but this increase was not robust, as we could only detect the change using the highly sensitive DAB iron staining method (Figure 1C). Despite liver and spleen iron loading, the *Fpn1^Tek/Tek^* mice were anemic with decreased serum iron levels and Tf saturation (Figure 1D, 1E, Figure 2A, 2B, Table S2 in File S1), suggesting that *Fpn1* deficiency impairs iron absorption and the mobilization of iron stores for erythropoiesis. Next, we measured gene expression in the livers of these mice (Figure 2C–E). Surprisingly, *Hamp1* mRNA was barely detectable in the *Fpn1^Tek/Tek^* mice (Figure 2C), despite liver iron loading.

Even more surprisingly, the mRNA levels of *Bmp6*, a potent stimulator of *Hamp1* expression typically expressed in abundance during iron overload, were significantly lower in the *Fpn1^Tek/Tek^* mice (Figure 2D), despite liver iron overload. This result suggests that factors other than liver iron level regulate *Bmp6* expression in these mice. As expected given their anemia, the *Fpn1^Tek/Tek^* mice had significantly higher erythropoietin (*Epo*) mRNA levels in the liver and kidneys (Figure 2E and data not shown). Given that p-Smad1/5/8 [8], [16], p-Erk1/2 [27] and p-Stat3 [32] are transcription factors that regulate *Hamp1* expression, we used western blot analysis to measure these proteins in liver lysates. The levels of p-Smad1/5/8 and p-Erk1/2—but not p-Stat3, a transcription factor implicated in inflammation-mediated stimulation of *Hamp1* expression—were reduced in the *Fpn1^Tek/Tek^* mice (Figure 2F, 2G), consistent with decreased *Hamp1* levels. Taken together, these data suggest that liver iron levels are not the sole factor involved in regulating *Bmp6* and *Hamp1* expression.

### Factors other than liver iron play a role in *Bmp6* down-regulation

**Figure 3:**
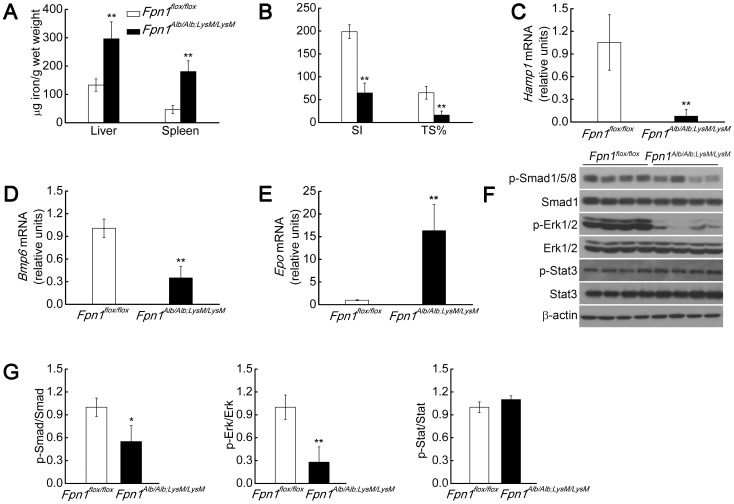
*Bmp6* and *Hamp1* expression decreases in young *Fpn1^Alb/Alb;LysM/LysM^* mice despite liver iron loading. (A) Liver and spleen non-heme iron concentrations. (B) SI and TS% levels. (C–E) Liver mRNA levels of *Hamp1* (C), *Bmp6* (D) and *Epo* (E). (F) Liver p-Smad1/5/8, Smad1, p-Erk1/2, Erk1/2, p-Stat3, Stat3 and β-actin protein levels were measured in 3-week-old male *Fpn1^flox/flox^* and *Fpn1^Alb/Alb;LysM/LysM^* mice (n = 5 per group). (G) Summary of the results in (F), quantitated using densitometry. Summary data are presented as mean ± SD. *P<0.05; **P<0.01.

The growth of *Fpn1^Tek/Tek^* mice is retarded, and these mice develop severe anemia (Figure 1D, 1E). Because the majority of these mice do not live beyond three weeks, we used *Fpn1^Alb/Alb;LysM/LysM^* mice—which have the combined deletion of *Fpn1* in hepatocytes and macrophages—to examine *Hamp1* regulation. These mice had high levels of iron in the liver and spleen (Figure 3A). At 2–3 weeks of age, the *Fpn1^Alb/Alb;LysM/LysM^* mice were more susceptible to iron deficiency than the *Fpn1^flox/flox^* mice. When a heterozygous mother was fed an iron-abundant diet, the pups developed virtually no anemia phenotype (data not shown). In contrast, pups born from a mother with the *Fpn1^Alb/Alb;LysM/LysM^* genotype developed anemia (Table S3 in File S1), although the anemia was less severe than in the *Fpn1^Tek/Tek^* mice (Table S2 and S3 in File S1). After weaning, the anemia resolved, possibly reflecting sufficient absorption of iron from the iron-sufficient diet to meet iron demand [28]. We then characterized the phenotype of these anemic, 3-week-old *Fpn1^Alb/Alb;LysM/LysM^* mice. Similar to *Fpn1^Tek/Tek^* mice, the *Fpn1^Alb/Alb;LysM/LysM^* mice had increased liver and spleen iron levels, decreased levels of serum Tf-bound iron, decreased Tf saturation and *Hamp1* expression, and increased *Epo* mRNA levels (Figure 3). Despite having liver iron overload (Figure 3A), liver *Bmp6* expression was decreased in the *Fpn1^Alb/Alb;LysM/LysM^* mice (Figure 3D), consistent with the *Fpn1^Tek/Tek^* mice (Figure 2D). Moreover, the levels of liver p-Smad1/5/8 and p-Erk1/2—but not p-Stat3—were also reduced in the *Fpn1^Alb/Alb;LysM/LysM^* mice (Figure 3F, 3G). Taken together, the phenotype of the *Fpn1^Alb/Alb;LysM/LysM^* mice provides further evidence that *Bmp6* and *Hamp1* are down-regulated under conditions of increased liver iron levels. This finding was not the result of aberrant behavior of the *Fpn1^flox^* allele, as feeding *Fpn1^flox/flox^* mice an iron-rich diet increased their levels of liver iron and serum iron, increased *Hamp1* and *Bmp6* expression, and increased the levels of the downstream transcription factor p-Smad1/5/8 (data not shown).

### Signals derived from the driving force of high iron demand may underlie *Bmp6* down-regulation

**Figure 4:**
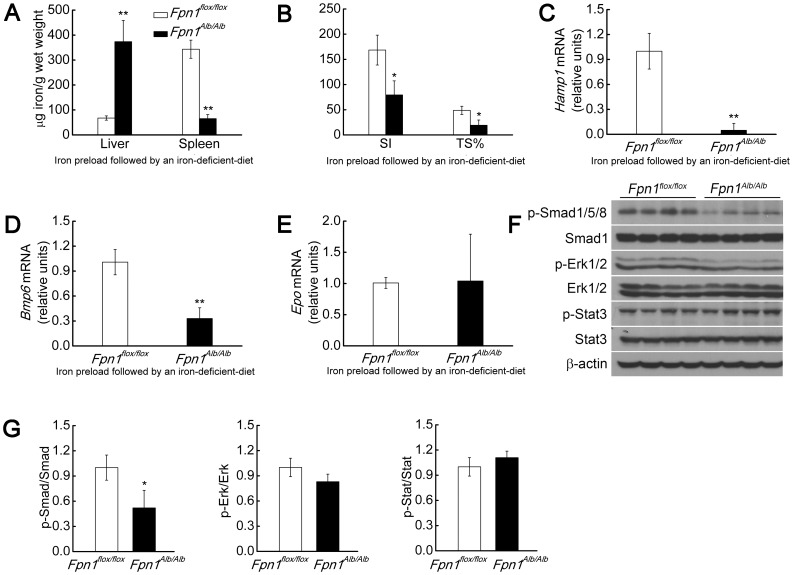
*Bmp6* and *Hamp1* expression decreases in the livers of *Fpn1^Alb/Alb^* mice with high iron demand. (A) Liver and spleen non-heme iron concentrations. (B) SI and TS%. (C–E) Liver mRNA levels of *Hamp1* (C), *Bmp6* (D), *Epo* (E). and (F) Liver p-Smad1/5/8, Smad1, p-Erk1/2, Erk1/2, p-Stat3, Stat3 and β-actin protein levels were measured in 3-week-old male *Fpn1^flox/flox^* and *Fpn1^Alb/Alb^* mice fed an iron-rich diet for one week, and then transferred to an iron-deficient diet for one month (n = 5 per group). (G) Summary of the results in (F), quantitated using densitometry. Summary data are presented as mean ± SD. *P<0.05; **P<0.01.

The aforementioned experiments using *Fpn1^Tek/Tek^* and *Fpn1^Alb/Alb;LysM/LysM^* mice suggest that factors other than liver iron levels regulate the expression of *Bmp6*. Because Tf-bound iron [23], anemia, Epo, and erythropoietic activity [5], [33]–[34] are all factors that influence *Hamp1* expression, we hypothesized that some or all of these factors might be involved in regulating *Bmp6* expression in our mouse models. To dissociate the effect of anemia, we established a liver iron-loaded mouse model that did not develop anemia. We first fed 3-week-old *Fpn1^flox/flox^* and *Fpn1^Alb/Alb^* mice an iron-rich diet for one week; the mice were then fed an iron-deficient diet for one month (see Methods). Our prediction was that after changing to an iron-deficient diet, iron would be mobilized from both the *Fpn1*-intact hepatocytes and macrophages in the *Fpn1^flox/flox^* mice, but would accumulate in the *Fpn1*-deficient hepatocytes in the *Fpn1^Alb/Alb^* mice. As we expected, compared to the *Fpn1^flox/flox^* mice, the *Fpn1^Alb/Alb^* mice had a 5-fold higher level of liver iron and lower spleen iron levels (Figure 4A). The *Fpn1^Alb/Alb^* mice showed no overt signs of anemia (Table S4 in File S1), suggesting that the pre-loaded iron could be mobilized and used. The *Fpn1^Alb/Alb^* mice also had decreased levels of serum Tf-bound iron, decreased Tf saturation (Figure 4B), and decreased *Hamp1* and *Bmp6* expression (Figure 4C, 4D). Consistent with their lack of anemia, *Epo* expression was not decreased in the *Fpn1^Alb/Alb^* mice (Figure 4E). Consistent with decreased liver *Bmp6* expression (Figure 4D), p-Smad1/5/8 levels were decreased in the *Fpn1^Alb/Alb^* mice (Figure 4F, 4G); in contrast, the levels of p-Erk1/2 and p-Stat3 were not significantly different between the *Fpn1^Alb/Alb^* and *Fpn1^flox/flox^* mice (Figure 4F, 4G). Thus, we surmised that the decreased *Hamp1* expression in *Fpn1^Alb/Alb^* mice was not due to the effects of anemia or increased *Epo* expression [35], but rather to decreased *Bmp6* expression and/or decreased serum levels of Tf-bound iron.

*Bmp6* expression is generally correlated with liver iron content [10]–[11]. Our observation that *Fpn1^Alb/Alb^* mice had 5-fold higher levels of liver iron but lower *Bmp6* expression levels compared to *Fpn1^flox/flox^* mice (Figure 4A, 4D) provides further evidence that liver iron levels are not the sole factor regulating *Bmp6* expression. Although the *Fpn1^Alb/Alb^* mice were not overtly anemic (Table S4 in File S1) and did not differ significantly from *Fpn1^flox/flox^* with respect to *Epo* expression (Figure 4E), they still had a large decrease in *Bmp6* expression relative to severely anemic *Fpn1^Tek/Tek^* mice (compare Figure 2D and Figure 4D). We therefore predicted that factors other than anemia and Epo regulate *Bmp6* expression in these mice. Because *Fpn1* was deleted in their hepatocytes, the *Fpn1^Alb/Alb^* mice had impaired liver iron mobilization, as indicated by their increased liver iron levels and decreased Tf-bound iron levels (Figure 4A, 4B). We interpreted the decreased levels of spleen iron in the *Fpn1^Alb/Alb^* mice (relative to the *Fpn1^flox/flox^* mice) as evidence of much stronger iron mobilization from other tissues to meet the higher iron demand in *Fpn1^Alb/Alb^* mice (Figure 4A). Based on our results obtained from the *Fpn1^Tek/Tek^* mice and actively growing *Fpn1^Alb/Alb;LysM/LysM^* mice (Figure 2, Figure 3), we hypothesized that signals arising from the driving force of high iron demand mediate *Bmp6* regulation, irrespective of liver iron. This prediction was supported by results obtained from non-anemic adult *Fpn1^flox/flox^* and *Fpn1^Alb/Alb;LysM/LysM^* mice. Given that both iron mobilization and iron recycling were impaired in the *Fpn1^Alb/Alb;LysM/LysM^* mice, we postulated that these mice have a much stronger driving force for absorbing iron to meet the body's demands [28].

To test this notion, we first measured *Bmp6* expression in adult *Fpn1^flox/flox^* and *Fpn1^Alb/Alb;LysM/LysM^* mice. Although *Fpn1^Alb/Alb;LysM/LysM^* mice have higher liver iron levels than control *Fpn1^flox/flox^* mice, their *Bmp6* expression level was not increased, suggesting blunted *Bmp6* expression (Figure S1A). Furthermore, when the adult *Fpn1^flox/flox^* and *Fpn1^Alb/Alb;LysM/LysM^* mice were fed an iron-deficient diet for two months, the *Fpn1^Alb/Alb;LysM/LysM^* mice had significantly lower *Bmp6* expression relative to *Fpn1^flox/flox^* mice, despite liver iron loading (Figure S1B). Based on these findings, we speculated that signals arising from the driving force of high iron demand might underlie the decrease in *Bmp6* expression, irrespective of liver iron loading. However, we could not exclude the possible involvement of other hepatic cell types (such as sinusoidal endothelial cells) in regulating *Bmp6* expression [36]–[37].

### Serum Tf-bound iron regulates *Hamp1* expression via p-Smad1/5/8 without affecting *Bmp6* expression

**Figure 5:**
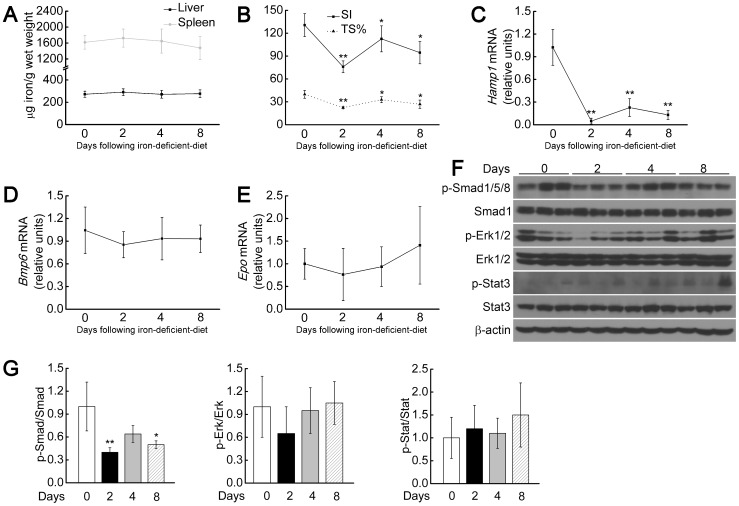
Changes in serum Tf-bound iron levels are consistent with p-smad1/5/8 and *Hamp1* levels in *Fpn1^Alb/Alb;LysM/LysM^* mice given an iron-deficient diet. (A) Time course of liver and spleen non-heme iron concentrations after switching to an iron-deficient diet. (B) Time course of SI and TS% levels. (C–E) Time course of liver mRNA levels of *Hamp1* (C), *Bmp6* (D), *Epo* (E). (F) Time course of liver p-Smad1/5/8, Smad1, p-Erk1/2, Erk1/2, p-Stat3, Stat3 and β-actin protein levels measured in 2-month-old male *Fpn1^Alb/Alb;LysM/LysM^* mice fed an iron-deficient diet for 0, 2, 4 or 8 days (n = 5 mice per time point). (G) Summary of the results in (F), quantitated using densitometry. Summary data are presented as mean ± SD. *P<0.05; **P<0.01.

Because the levels of Tf-bound iron were correlated with *Bmp6* expression in the *Fpn1^Tek/Tek^* (Figure 2B, 2D), *Fpn1^Alb/Alb;LysM/LysM^* (Figure 3B, 3D), and *Fpn1^Alb/Alb^* (Figure 4B, 4D) mice, we hypothesized that Tf-bound iron may regulate *Bmp6* expression in our animal models. To dissect the effect of Tf-bound iron on *Bmp6* expression, we first placed adult *Fpn1^flox/flox^* mice on a short-term iron-deficient diet. These mice developed decreased levels of liver iron, serum Tf-bound iron, and Tf saturation, decreased *Bmp6* and *Hamp1* mRNA, and decreased p-Smad1/5/8 levels (Figure S2). We next placed *Fpn1^Alb/Alb;LysM/LysM^* mice on a short-term iron-deficient diet. Unlike the *Fpn1^flox/flox^* mice, the *Fpn1^Alb/Alb;LysM/LysM^* mice (which have hepatocyte- and macrophage-specific *Fpn1* deletion) had relatively stable liver and spleen iron levels (Figure 5A); however, circulating Tf-bound iron levels and Tf saturation decreased immediately upon starting the iron-deficient diet (Figure 5B). Nevertheless, these mice did not develop obvious signs of anemia (Table S5 in File S1). Liver *Hamp1* expression was dramatically down-regulated in these mice (Figure 5C), whereas both *Bmp6* and *Epo* expression had not reached significance on the iron-deficient diet (Figure 5D, 5E). Finally, the levels of p-Smad1/5/8 decreased in parallel with the Tf-bound iron levels and liver *Hamp1* expression, whereas p-Erk1/2 and p-Stat3 levels were unchanged (Figure 5F, 5G). These results suggest that serum Tf-bound iron regulates *Hamp1* expression via p-Smad1/5/8 with no detectable effect on *Bmp6* expression.

To further investigate the role of Tf-bound iron in *Bmp6* and *Hamp1* regulation, we performed two additional experiments. First, we used a previously characterized mouse model [38]. *Tf^hpx/hpx^ Hjv^−/−^* mice lack both Tf and Hjv expression. We previously reported that treating *Tf^hpx/hpx^ Hjv^+/+^* and *Tf^hpx/hpx^ Hjv^−/−^* mice with Tf normalized the hemoglobin levels of both mouse models; in contrast, *Hamp1* expression increased robustly in the *Tf^hpx/hpx^ Hjv^+/+^* mice only (but not in the *Tf^hpx/hpx^ Hjv^−/−^* mice). *Bmp6* expression was not affected by Tf treatment in either genotype [38]. To determine whether any signaling pathways in these mice were affected by Tf treatment, we measured the protein levels of several transcription factors in these mice and found increased p-Smad1/5/8 levels in the Tf-treated *Tf^hpx/hpx^ Hjv^+/+^* mice only (Figure S3A). Neither the p-Erk1/2 nor p-Stat3 levels were affected by Tf treatment in either genotype (Figure S3B, S3C). These results provide further evidence that Tf-bound iron regulates *Hamp1* expression via p-Smad1/5/8.

Second, to disassociate the relationship between Tf-bound iron and iron demand status, *Fpn1^flox/flox^* mice were injected intraperitoneally with 10 mg holo-Tf, and then analyzed the following day. Consistent with increased Tf-bound iron levels, *Hamp1* expression and p-Smad1/5/8 levels increased approximately 2-fold in the holo-Tf-injected mice (Figure S4), despite having no increase in *Bmp6* expression [38]. These results suggest that Tf-bound iron did not play a role in *Bmp6* regulation in these mice. Thus, we conclude that Tf-bound iron regulates *Hamp1* expression via p-Smad1/5/8 without affecting *Bmp6* expression, which is consistent with previous reports [23].

In conclusion, factors other than liver iron levels regulated *Bmp6* expression in our experiments. We postulate that the *Bmp6*-regulating signals arose from the driving force of high iron demand. However, we cannot exclude the possibility that the iron status of other hepatic cell types might also influence *Bmp6* regulation. Our results also support previous findings that Tf-bound iron regulates *Hamp1* expression via p-Smad1/5/8 without affecting *Bmp6* expression.

## Discussion

The liver is the major organ for storing iron. Liver iron levels reflect the whole-body iron status and correlate nicely with *Hamp1* expression [10]. Under physiological conditions, iron overload increases *Hamp1* expression, restricting intestinal iron absorption and iron release from iron storage sites; on the other hand, iron deficiency has the opposite effect. However, because *Hamp1* deficiency is a known feature in mouse models of β-thalassemia [39] and hypotransferrinemia [22] (both of which are conditions associated with iron overload), other factors must be involved in *Hamp1* regulation. Tf-bound iron [23], [40] and Epo [34] are known regulators of *Hamp1* expression. However, because these—and other—regulatory factors are often present together, we used conditional *Fpn1*-knockout mouse models that have relatively stable tissue iron levels, and we subjected these animals to various stressors to dissect the roles and interactions of various factors in regulating *Hamp1* expression.

Both *Fpn1^Tek/Tek^* and actively growing *Fpn1^Alb/Alb;LysM/LysM^* mice developed an intriguing phenotype, namely decreased *Hamp1* expression (Figure 2C, Figure 3C) in the context of liver iron loading (Figure 2A, Figure 3A). This phenotype has been observed previously in mouse models of hypotransferrinemia and other diseases [22], [39]. However, a clear difference between hypotransferrinemic mice and our *Fpn1* mouse models is that *Bmp6* expression was decreased—not increased—in our mice (Figure 2D, Figure 3D). To our knowledge, this is the first report of such a phenotype, and it suggests that factors other than liver iron must play a role in controlling *Bmp6* expression in these mice. However, it should be noted that decreased *Bmp6* expression relative to liver iron levels has been reported in β-thalassemic mice [41].

We first hypothesized that the decreased *Bmp6* expression in our *Fpn1* mouse models might be due to anemia and/or Epo. However, this seemed unlikely, given that *Fpn1^Alb/Alb^* mice (which were first pre-loaded with iron, and then transferred to an iron-poor diet to drive tissue iron mobilization) had liver iron loading and decreased *Bmp6* and *Hamp1* expression, yet developed no significant signs of anemia (Table S4 in File S1) or changes in *Epo* expression compared to control *Fpn1^flox/flox^* mice (Figure 4E). We then theorized that decreased *Bmp6* expression could be attributed directly to the lack of *Fpn1* expression. However, we found no significant difference in *Bmp6* expression between adult *Fpn1^flox/flox^* and *Fpn1^Alb/Alb^* mice that were fed an iron-rich diet (data not shown). Thus, we could exclude a direct role for Fpn1 in regulating *Bmp6* expression. When *Fpn1^flox/flox^* and *Fpn1^Alb/Alb^* mice were pre-loaded with iron and then placed on an iron-deficient diet, we reasoned that iron demand would be high in the *Fpn1^Alb/Alb^* mice, given that iron mobilization from their hepatocytes was impaired. Therefore, we inferred that the signal for *Bmp6* down-regulation in *Fpn1^Alb/Alb^* mice might arise from the driving force of high iron demand. This notion was validated in adult *Fpn1^Alb/Alb;LysM/LysM^* mice that were fed an iron-replete or iron-deficient diet (Figure S1). Additionally, if the signal for iron demand indeed affects *Bmp6* expression, the phenotype of hypotransferrinemic (*Tf^hpx/hpx^*) mice must be rectified, given that these mice are profoundly anemic yet still have increased *Bmp6* expression [22]. However, the increased *Bmp6* expression in these mice could be explained by their extremely high liver iron levels, which could overwhelm the effect of high iron demand on *Bmp6* regulation.

Moreover, mice with phenylhydrazine-induced hemolysis have high iron demand, yet still have high *Bmp6* expression, which can also be explained by an increase in liver iron levels following phenylhydrazine treatment [42]. Similarly, β-thalassemic mice have high iron demand and increased *Bmp6* expression, although the magnitude of the increase in *Bmp6* expression was not as large as expected [41]–[42]. Thus, we hypothesize that the factors that signal iron overload and erythroid iron demand have opposing and competing effects on *Bmp6* expression in these contexts. Of course, we cannot exclude the possibilty that other factors might play a role in altering *Bmp6* expression in these model systems. For example, hepatic sinusoidal endothelial cells are a more robust source of *Bmp6* expression than hepatic stellate cells, Kupffer cells, and hepatocytes [36]–[37]. Finally, the iron status of hepatic non-parenchymal cells might affect *Bmp6* expression in our mouse models, and this possibility merits further study.

Because Tf-bound iron levels can reflect the balance between iron supply and erythroid demand, we also investigated the role of serum Tf-bound iron in regulating *Bmp6* and/or *Hamp1* expression. Placing adult *Fpn1^Alb/Alb;LysM/LysM^* mice on an iron-deficient diet had no short-term effect on liver or spleen iron concentrations, yet Tf-bound iron levels were decreased significantly, as were both *Hamp1* expression and p-Smad1/5/8 levels (Figure 5). However, no significant change was found with respect to *Bmp6* expression (Figure 5D), although a slight trend towards decreased expression was noted, which may reflect the higher iron demand status. Because the change in *Bmp6* was negligible compared to the significant changes in both p-Smad1/5/8 and *Hamp1* mRNA, these results suggest that Tf-bound iron down-regulated *Hamp1* expression primarily via Smad1/5/8 phosphorylation.

To investigate further the role of Tf-bound iron in *Bmp6* regulation, we also treated *Tf^hpx/hpx^ Hjv^+/+^* mice with Tf. This treatment increased the levels of p-Smad1/5/8 (Figure S3) and *Hamp1* mRNA, but had no effect on *Bmp6* expression [38]. This finding was confirmed in *Fpn1^flox/flox^* mice that were injected with holo-Tf (Figure S4). Together, these data do not necessarily support a role for Tf-bound iron in *Bmp6* regulation, but they do reinforce the previous report that Tf-bound iron regulates *Hamp1* via p-Smad1/5/8 [23].

While the Smad pathway plays a central role in regulating *Hamp1* expression, p-Erk1/2 has also been implicated in mediating *Hamp1* expression [27], [40]. However, with the exception of 13–15-day-old anemic *Fpn1^Tek/Tek^* mice and 3-week-old *Fpn1^Alb/Alb;LysM/LysM^* mice (Figure 2F and 3F), no significant changes in p-Erk1/2 levels were observed in our mouse models, regardless of whether they were subjected to changes in dietary iron or injected with holo-Tf (Figure 4F, Figure 5F, , S3B, S4F). Overall, our findings do not support a role for p-Erk1/2 in *Hamp1* regulation in this context. However, at this time we cannot explain the significant decrease in p-Erk1/2 levels in the anemic *Fpn1^Tek/Tek^* and *Fpn1^Alb/Alb;LysM/LysM^* mice. It is possible that p-Erk1/2 plays a more important role in *Hamp1* expression in early development or during overt anemia. Furthermore, in some cases, the variability in the p-Erk1/2 signal was too large to allow conclusive results to be drawn (Figure 5F, Figure S3B, S4F). Studying Erk1/2-deficient mice may be a more definitive approach for determining whether Erk1/2 plays a role in regulating *Hamp1* expression.

In conclusion, our results suggest that factors other than liver iron can regulate *Bmp6* expression. The *Bmp6* down-regulation observed in our *Fpn1*-deficient mice was not due to the effect of Tf-bound iron, anemia, or Epo expression. We speculate that the signals that underlie the decreased *Bmp6* expression in our mouse models arose from the driving force of high iron demand. In addition, our results support previous findings that serum Tf-bound iron regulates *Hamp1* expression via p-Smad1/5/8 without affecting *Bmp6* expression. Given that other factors such as Epo expression, inflammatory factors, and reactive oxygen species have been reported to play important roles in regulating *Hamp1* expression, the interplay between these factors under various physiological conditions warrant further study.

## Supporting Information

Figure S1 ***Bmp6*** **expression is down-regulated in mice with high-iron demand.** Liver mRNA levels of *Bmp6* were measured in 2-month-old male *Fpn1^flox/flox^* and *Fpn1^Alb/Alb;LysM/LysM^* mice without anemia (A) or in 2-month-old *Fpn1^flox/flox^* and *Fpn1^Alb/Alb;LysM/LysM^* mice that were fed an iron-deficient diet for two months (B). n = 5 per group. Data are presented as mean ± SD. *P<0.05.
(TIF)

Figure S2 **Liver iron, serum Tf-bound iron and** ***Hamp1*** **and** ***Bmp6*** **expression levels are decreased in adult** ***Fpn1^flox/flox^*** **mice placed on a short-term iron-deficient diet.** (A) Liver and spleen non-heme iron concentrations. (B) SI and TS% levels. (C) Liver mRNA levels of *Hamp1*, (D) *Bmp6* and (E) *Epo*. (F) Liver p-Smad1/5/8, Smad1, p-Erk1/2, Erk1/2, p-Stat3, Stat3 and β-actin protein levels were measured in 2-month-old male *Fpn1^flox/flox^* mice fed an AIN-76A (iron-deficient) diet for 0, 2, 4, or 8 days (n = 5 per group). Summary data are presented as mean ± SD. *P<0.05; **P<0.01.
(TIF)

Figure S3 **Tf stimulates** ***Hamp1*** **expression via p-Smad1/5/8.** Mice deficient in Tf (*Tf^hpx/hpx^ Hjv^+/+^*) or Tf and Hjv (*Tf^hpx/hpx^ Hjv^−/−^*) were treated with 10 mg Tf or an equivalent volume of PBS every other day for two weeks; the organs were then harvested for analysis. Protein levels of (A) p-Smad1/5/8 and Smad1, (B) p-Erk1/2 and Erk1/2, (C) p-Stat3 and Stat3 were measured by western blot analysis of lysates prepared from the harvested livers; β-actin was measured as a loading control. The blots were analyzed by densitometry, and the ratios of phosphorylated protein to total protein are expressed graphically in the right panels. Summary data are presented as mean ± SD. *P<0.05.
(TIF)

Figure S4 **Holo-Tf supplementation regulates** ***Hamp1*** **through p-smad1/5/8 without influencing** ***Bmp6*** **expression.** (A) Liver and spleen non-heme iron concentrations. (B) SI and TS% levels. (C) Liver mRNA levels of *Hamp1*, (D) *Bmp6* and (E) *Epo*. (F) Liver p-Smad1/5/8, Smad1, p-Erk1/2, Erk1/2, p-Stat3, Stat3, and β-actin protein levels were measured in 2-month-old male *Fpn1^flox/flox^* mice that were injected with 10 mg holo-Tf in PBS (or an equal volume of PBS) and then fed *ad libitum* overnight to facilitate saturation of Tf with iron (n = 5 per group). Summary data are presented as mean ± SD. *P<0.05; **P<0.01.
(TIF)

File S1 **Tables S1–S5. Table S1. Sequences of primers. Table S2. Hematologic parameters of** ***Fpn1^flox/flox^*** **and** ***Fpn1^Tek/Tek^*** **mice.** Hematologic parameters were measured in 13–15-day-old male *Fpn1^flox/flox^* and *Fpn1^Tek/Tek^* mice. RBCs, Red Blood Cells; HGB, hemoglobin; HCT, hematocrit; MCV, mean corpuscular volume; MCH, mean corpuscular hemoglobin; MCHC, mean corpuscular hemoglobin concentration. Data are presented as mean ± SD. *P<0.05; **P<0.01. **Table S3. Hematologic parameters of three-week-old** ***Fpn1^flox/flox^*** **and** ***Fpn1^Alb/Alb;LysM/LysM^*** **mice.** Hematologic parameters were measured in 3-week-old *Fpn1^flox/flox^* and *Fpn1^Alb/Alb;LysM/LysM^* mice. Data are presented as mean ± SD. P<0.05; **P<0.01. **Table S4. Hematologic parameters of** ***Fpn1^flox/flox^*** **and** ***Fpn1^Alb/Alb^*** **mice preloaded with iron then maintained on an iron-deficient diet for one month.** Three-week-old male *Fpn1^flox/flox^* and *Fpn1^Alb/Alb^* mice were fed an iron-rich diet (8.3 g of carbonyl iron/kg) for one week, and then transferred to an iron-deficient diet (0.9 mg iron/kg) for one month. Blood was harvested for hematologic parameters analysis. Results are presented as mean ± SD. *P<0.05; **P<0.01. **Table S5. Hematologic parameters of** ***Fpn1^Alb/Alb;LysM/LysM^*** **mice maintained short-term on an iron-deficient diet.** Two-month-old male *Fpn1^Alb/Alb;LysM/LysM^* mice were fed an AIN-76A (iron-deficient) diet (0.9 mg iron/kg) for 0, 2, 4, or 8 days (n = 5 per group). Blood was then harvested for hematologic parameter analysis. Blood parameters of *Fpn1^flox/flox^* mice at day 0 were measured as a control. Results are presented as mean ± SD. *P<0.05; **P<0.01.
(DOC)
