# Supplementary figures and images for: Bmp6 Expression Can Be Regulated Independently of Liver Iron in Mice
Source: PLoS One. 2014 Jan 13;9(1):e84906. doi: 10.1371/journal.pone.0084906 (PMC3890292; doi:10.1371/journal.pone.0084906)

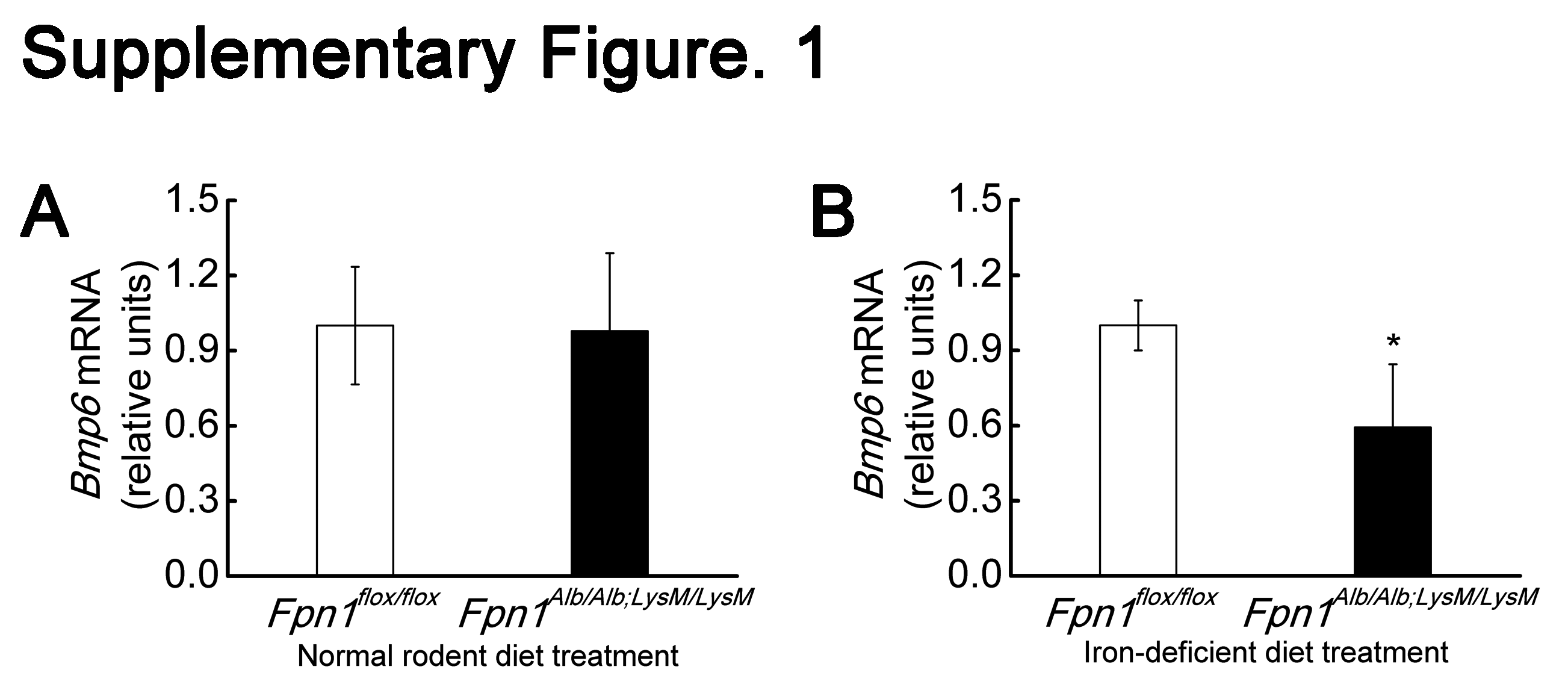

Supplement: Figure S1 — Bmp6 expression is down-regulated in mice with high-iron demand. Liver mRNA levels of Bmp6 were measured in 2-month-old male Fpn1flox/flox and Fpn1Alb/Alb;LysM/LysM mice without anemia (A) or in 2-month-old Fpn1flox/flox and Fpn1Alb/Alb;LysM/LysM mice that were fed an iron-deficient diet for two months (B). n = 5 per group. Data are presented as mean ± SD. *P<0.05. (TIF) [file pone.0084906.s001.tif]

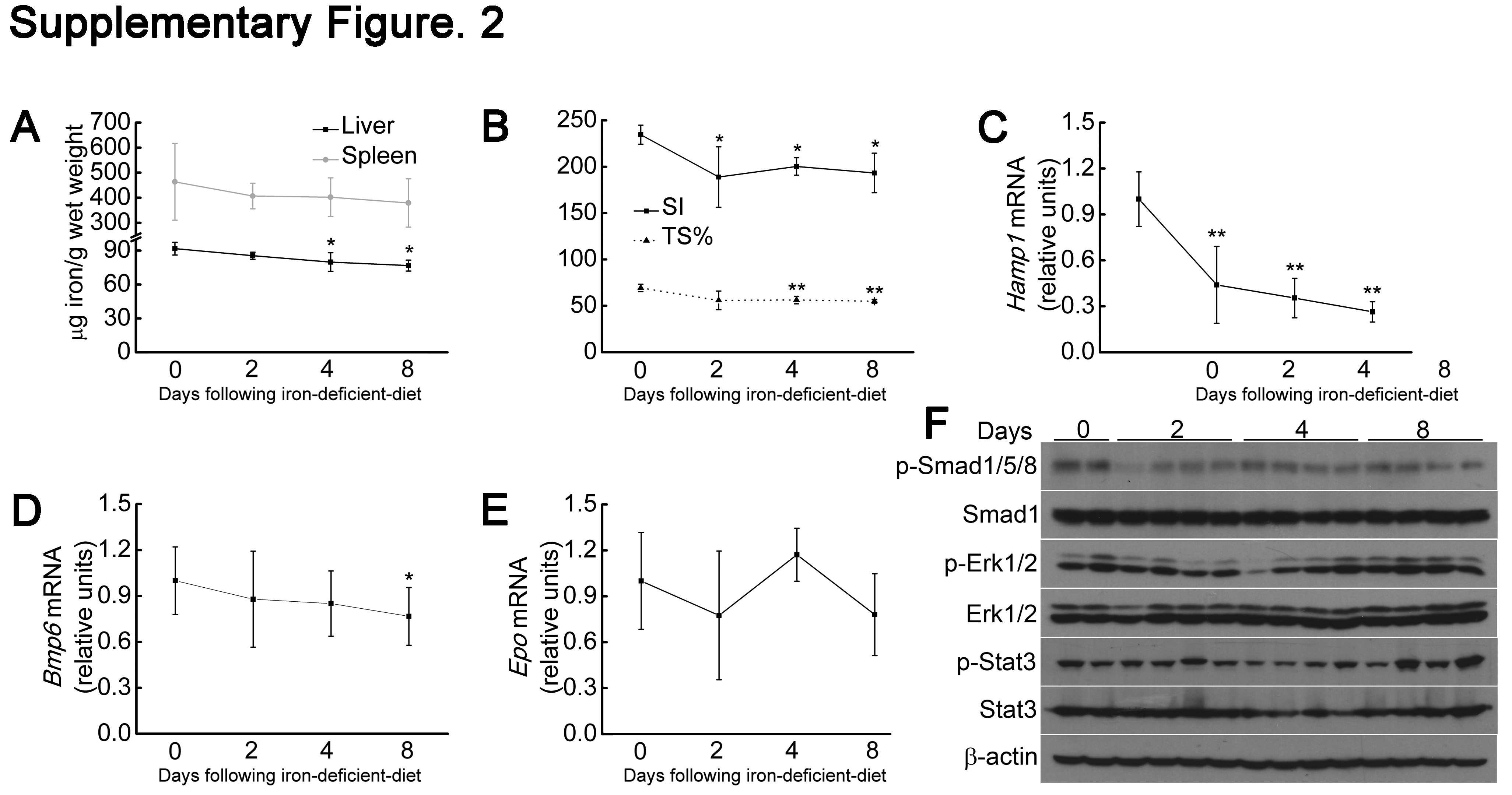

Supplement: Figure S2 — Liver iron, serum Tf-bound iron and Hamp1 and Bmp6 expression levels are decreased in adult Fpn1flox/flox mice placed on a short-term iron-deficient diet. (A) Liver and spleen non-heme iron concentrations. (B) SI and TS% levels. (C) Liver mRNA levels of Hamp1, (D) Bmp6 and (E) Epo. (F) Liver p-Smad1/5/8, Smad1, p-Erk1/2, Erk1/2, p-Stat3, Stat3 and β-actin protein levels were measured in 2-month-old male Fpn1flox/flox mice fed an AIN-76A (iron-deficient) diet for 0, 2, 4, or 8 days (n = 5 per group). Summary data are presented as mean ± SD. *P<0.05; **P<0.01. (TIF) [file pone.0084906.s002.tif]

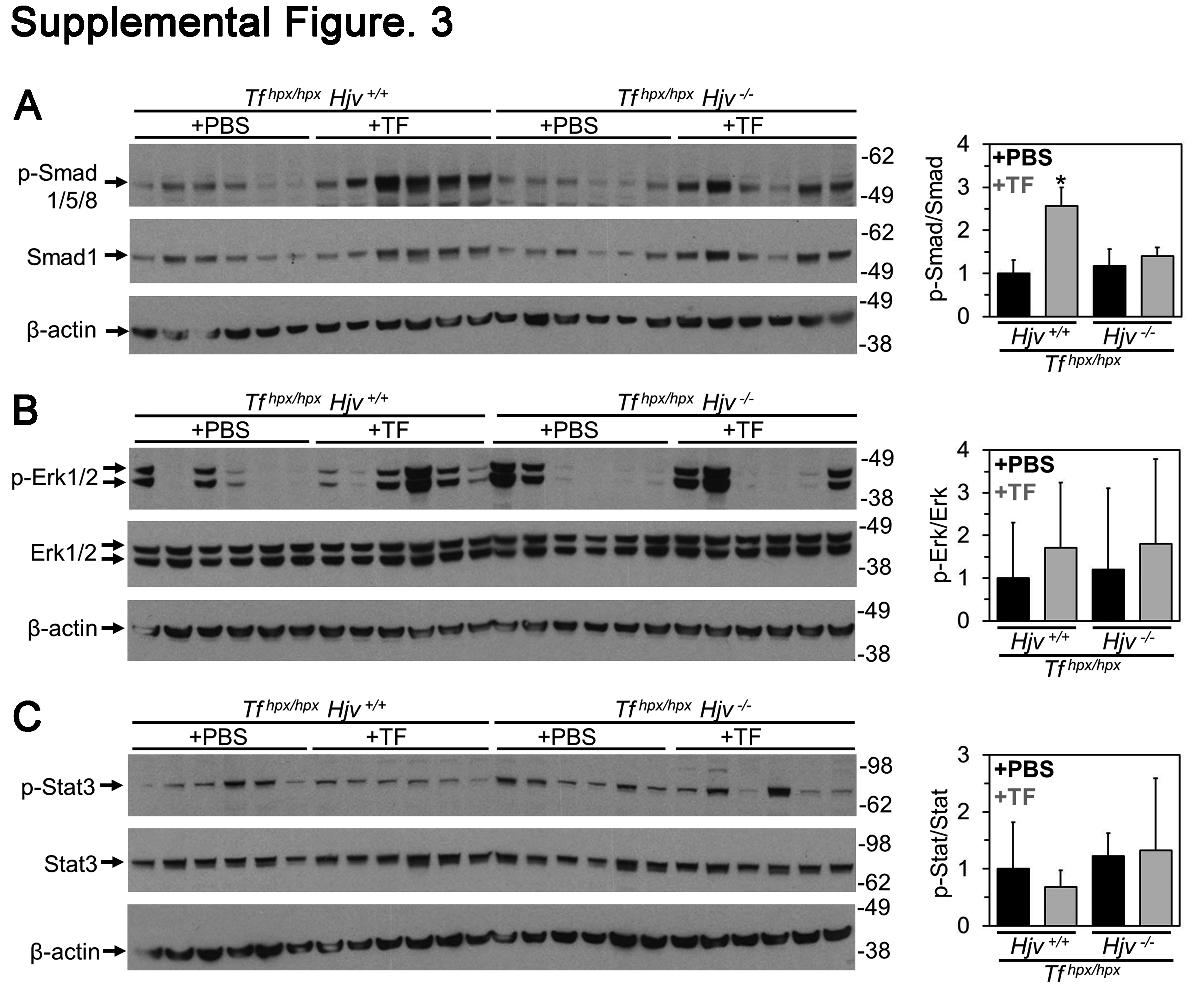

Supplement: Figure S3 — Tf stimulates Hamp1 expression via p-Smad1/5/8. Mice deficient in Tf (Tfhpx/hpx Hjv+/+) or Tf and Hjv (Tfhpx/hpx Hjv−/−) were treated with 10 mg Tf or an equivalent volume of PBS every other day for two weeks; the organs were then harvested for analysis. Protein levels of (A) p-Smad1/5/8 and Smad1, (B) p-Erk1/2 and Erk1/2, (C) p-Stat3 and Stat3 were measured by western blot analysis of lysates prepared from the harvested livers; β-actin was measured as a loading control. The blots were analyzed by densitometry, and the ratios of phosphorylated protein to total protein are expressed graphically in the right panels. Summary data are presented as mean ± SD. *P<0.05. (TIF) [file pone.0084906.s003.tif]

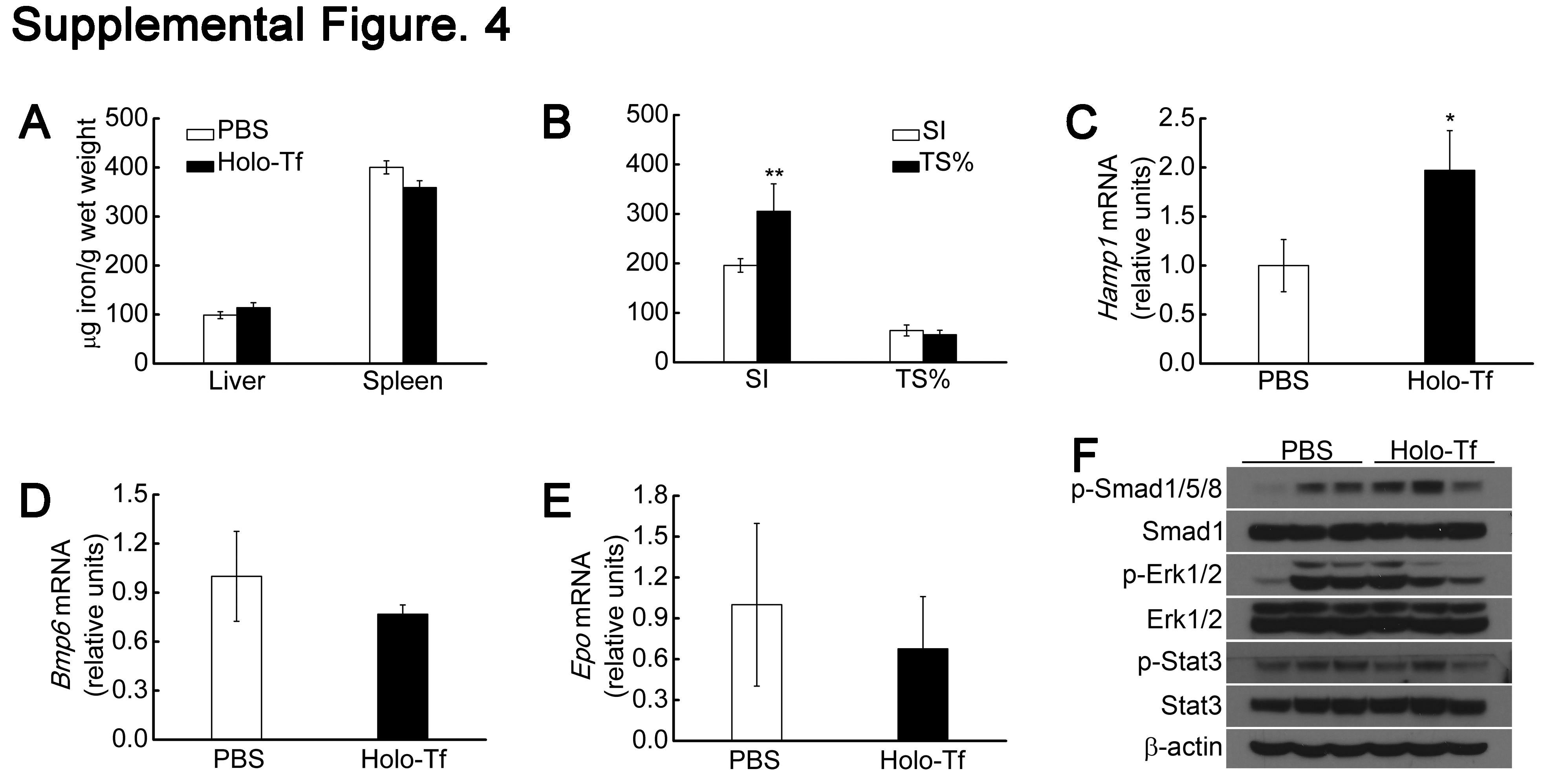

Supplement: Figure S4 — Holo-Tf supplementation regulates Hamp1 through p-smad1/5/8 without influencing Bmp6 expression. (A) Liver and spleen non-heme iron concentrations. (B) SI and TS% levels. (C) Liver mRNA levels of Hamp1, (D) Bmp6 and (E) Epo. (F) Liver p-Smad1/5/8, Smad1, p-Erk1/2, Erk1/2, p-Stat3, Stat3, and β-actin protein levels were measured in 2-month-old male Fpn1flox/flox mice that were injected with 10 mg holo-Tf in PBS (or an equal volume of PBS) and then fed ad libitum overnight to facilitate saturation of Tf with iron (n = 5 per group). Summary data are presented as mean ± SD. *P<0.05; **P<0.01. (TIF) [file pone.0084906.s004.tif]
