# Supplementary material for: Bmp6 Expression Can Be Regulated Independently of Liver Iron in Mice
Source: PLoS One. 2014 Jan 13;9(1):e84906. doi: 10.1371/journal.pone.0084906 (PMC3890292; doi:10.1371/journal.pone.0084906)
Supplement: File S1 — Tables S1–S5. Table S1. Sequences of primers. Table S2. Hematologic parameters of Fpn1flox/flox and Fpn1Tek/Tek mice. Hematologic parameters were measured in 13–15-day-old male Fpn1flox/flox and Fpn1Tek/Tek mice. RBCs, Red Blood Cells; HGB, hemoglobin; HCT, hematocrit; MCV, mean corpuscular volume; MCH, mean corpuscular hemoglobin; MCHC, mean corpuscular hemoglobin concentration. Data are presented as mean ± SD. *P<0.05; **P<0.01. Table S3. Hematologic parameters of three-week-old Fpn1flox/flox and Fpn1Alb/Alb;LysM/LysM mice. Hematologic parameters were measured in 3-week-old Fpn1flox/flox and Fpn1Alb/Alb;LysM/LysM mice. Data are presented as mean ± SD. P<0.05; **P<0.01. Table S4. Hematologic parameters of Fpn1flox/flox and Fpn1Alb/Alb mice preloaded with iron then maintained on an iron-deficient diet for one month. Three-week-old male Fpn1flox/flox and Fpn1Alb/Alb mice were fed an iron-rich diet (8.3 g of carbonyl iron/kg) for one week, and then transferred to an iron-deficient diet (0.9 mg iron/kg) for one month. Blood was harvested for hematologic parameters analysis. Results are presented as mean ± SD. *P<0.05; **P<0.01. Table S5. Hematologic parameters of Fpn1Alb/Alb;LysM/LysM mice maintained short-term on an iron-deficient diet. Two-month-old male Fpn1Alb/Alb;LysM/LysM mice were fed an AIN-76A (iron-deficient) diet (0.9 mg iron/kg) for 0, 2, 4, or 8 days (n = 5 per group). Blood was then harvested for hematologic parameter analysis. Blood parameters of Fpn1flox/flox mice at day 0 were measured as a control. Results are presented as mean ± SD. *P<0.05; **P<0.01. (DOC) [file pone.0084906.s005.doc]

**Figure S1. *Bmp6* expression is down-regulated in mice with high-iron demand**. Liver mRNA levels of *Bmp6* were measured in 2-month-old male *Fpn1^flox/flox^* and *Fpn1^Alb/Alb;LysM/LysM^* mice without anemia (A) or in 2-month-old *Fpn1^flox/flox^* and *Fpn1^Alb/Alb;LysM/LysM^* mice that were fed an iron-deficient diet for two months (B). n=5 per group. Data are presented as mean ± SD. *****P<0.05.

**Figure S2. Liver iron, serum Tf-bound iron and *Hamp1* and *Bmp6* expression levels are decreased in adult *Fpn1^flox/flox^* mice placed on a short-term iron-deficient diet.** (A) Liver and spleen non-heme iron concentrations. (B) SI and TS% levels. (C) Liver mRNA levels of *Hamp1*, (D) *Bmp6* and (E) *Epo*. (F) Liver p-Smad1/5/8, Smad1, p-Erk1/2, Erk1/2, p-Stat3, Stat3 and β-actin protein levels were measured in 2-month-old male *Fpn1^flox/flox^* mice fed an AIN-76A (iron-deficient) diet for 0, 2, 4, or 8 days (n=5 per group). Summary data are presented as mean ± SD. *****P<0.05; ******P<0.01.

**Figure S3. Tf stimulates *Hamp1* expression via p-Smad1/5/8.** Mice deficient in Tf (*Tf^hpx/hpx^ Hjv^+/+^*) or Tf and Hjv (*Tf^hpx/hpx^ Hjv^-/-^*) were treated with 10 mg Tf or an equivalent volume of PBS every other day for two weeks; the organs were then harvested for analysis. Protein levels of (A) p-Smad1/5/8 and Smad1, (B) p-Erk1/2 and Erk1/2, (C) p-Stat3 and Stat3 were measured by western blot analysis of lysates prepared from the harvested livers; β-actin was measured as a loading control. The blots were analyzed by densitometry, and the ratios of phosphorylated protein to total protein are expressed graphically in the right panels. Summary data are presented as mean ± SD. *P<0.05.

**Figure S4.** **Holo-Tf supplementation regulates *Hamp1* through p-smad1/5/8 without influencing *Bmp6* expression.** (A) Liver and spleen non-heme iron concentrations. (B) SI and TS% levels. (C) Liver mRNA levels of *Hamp1*, (D) *Bmp6* and (E) *Epo*. (F) Liver p-Smad1/5/8, Smad1, p-Erk1/2, Erk1/2, p-Stat3, Stat3, and β-actin protein levels were measured in 2-month-old male *Fpn1^flox/flox^* mice that were injected with 10 mg holo-Tf in PBS (or an equal volume of PBS) and then fed *ad libitum* overnight to facilitate saturation of Tf with iron (n=5 per group). Summary data are presented as mean ± SD. *****P<0.05; ******P<0.01.

**Table S1. Sequences of the primers used for the qRT-PCR assays.**

| **Gene name** | **Forward primer** | **Reverse primer** |
| --- | --- | --- |
| *β-actin* | AAATCGTGCGTGACATCAAAGA | GCCATCTCCTGCTCGAAGTC |
| *Bmp6* | AACCTTTCTTATCAGCATTTACCA | GTGTCCAACAAAAATAGGTCAGAG |
| *Fpn1* | GTCGGCCAGATTATGACATTTG | ATTCCAACCGGAAATAAAACCA |
| *Hamp1* | GCACCACCTATCTCCATCAACA | TTCTTCCCCGTGCAAAGG |
| *Epo* | TCCCCCACGCCTCATCT | TTTCTGCCTCCTTGGCCTCTA |
| *Smad7* | TGGATGGCGTGTGGGTTTA | TGGCGGACTTGATGAAGATG |
| *Gdf-15* | ACCCCGGTGGTTCTTATGC | CAGGTCATCATAAGTCTGCAGTGA |
| *Tgfb1* | GAGCCCGAAGCGGACTACT | TTGCGGTCCACCATTAGCA |
| *Twsg1* | GTGCACAGTGGTTTACTTTGATGAC | CCATGGATTCGCAGGATATCTT |
| *Tmprss6* | ACGTGCATTTCACTGCCTAGAG | TGTTCTTCGTCACTGCCATTG |
| *Hjv* | CCAGGCTGAGGTGGACAATC | GTCGGTCGCCCCCATT |
| *Hfe* | TGTGAGGTGCATGAAGACAACAG | TCTTGCCCGTCATAACCATATCT |
| *Tek-Cre* | CGCATAACCAGTGAAACAGCATTGC | CCCTGTGCTCAGACAGAAATGAGA |

**Table S2. Hematologic parameters of the *Fpn1^flox/flox^* and *Fpn1^Tek/Tek^* mice.**

|  | ***Fpn1^flox/flox^*** (*n=5*) | ***Fpn1^Tek/Tek^*** (*n=5*) |
| --- | --- | --- |
| RBCs (10^12^/L) | 6.24±0.43 | 5.24±0.40 * |
| HGB (g/L) | 116.3±1.9 | 85.3±2.5 ** |
| HCT | 0.39±0.01 | 0.31±0.03 ** |
| MCV (fL) | 61.9±3.5 | 57.4±2.2 * |
| MCH (pg) | 18.7±1.1 | 16.2±0.5 * |
| MCHC (g/L) | 302.1±3.0 | 282.5±5.8 ** |

Hematologic parameters were measured in 13- 15-day-old male *Fpn1^flox/flox^* and *Fpn1^Tek/Tek^* mice. RBCs, Red Blood Cells; HGB, hemoglobin; HCT, hematocrit; MCV, mean corpuscular volume; MCH, mean corpuscular hemoglobin; MCHC, mean corpuscular hemoglobin concentration. Data are presented as mean ± SD. *****P<0.05; ******P<0.01.

**Table S3. Hematologic parameters of 3-week-old *Fpn1^flox/flox^* and *Fpn1^Alb/Alb;LysM/LysM^* mice.**

|  | ***Fpn1^flox/flox^*** (*n=5*) | ***Fpn1^Alb/Alb;LysM/LysM^*** (*n=5*) |
| --- | --- | --- |
| RBCs (10^12^/L) | 7.53±0.18 | 6.77±0.29 * |
| HGB (g/L) | 125.5±3.5 | 113.0±4.1 * |
| HCT | 0.46±0.01 | 0.41±0.02 * |
| MCV (fL) | 61.2±0.3 | 61.1±0.9 |
| MCH (pg) | 16.7±0.1 | 16.7±0.2 |
| MCHC (g/L) | 272.5±2.1 | 272.3±4.0 |

Hematologic parameters were measured in 3-week-old *Fpn1^flox/flox^* and *Fpn1^Alb/Alb;LysM/LysM^* mice. Data are presented as mean ± SD. P<0.05; ******P<0.01.

**Table S4. Hematologic parameters of *Fpn1^flox/flox^* and *Fpn1^Alb/Alb^* mice that were preloaded with iron and then fed an** iron-deficient diet for one month

|  | ***Fpn1^flox/flox^*** (*n=5*) | ***Fpn1^Alb/Alb^*** (*n=5*) |
| --- | --- | --- |
| RBCs (10^12^/L) | 10.28±1.16 | 9.99±0.57 |
| HGB (g/L) | 146.5±3.6 | 138.4±4.8 |
| HCT | 0.50±0.05 | 0.45±0.04 |
| MCV (fL) | 46.7±1.0 | 45.4±1.1 |
| MCH (pg) | 14.3±0.6 | 13.8±0.2 |
| MCHC (g/L) | 305.1±3.3 | 303.9±2.7 |

Three-week-old male *Fpn1^flox/flox^* and *Fpn1^Alb/Alb^* mice were fed an iron-rich diet (8.3 g of carbonyl iron/kg) for one week, and then transferred to an iron-deficient diet (0.9 mg iron/kg) for one month. Blood was harvested for hematologic parameters analysis. Results are presented as mean ± SD. *****P<0.05; ******P<0.01.

**Table S5. Hematologic parameters of *Fpn1^Alb/Alb;LysM/LysM^* mice that were fed an iron-deficient diet for the indicated number of days.**

|  | *Fpn^flox/flox^* | *Fpn1^Alb/Alb/LysM/LysM^* | | | |
| --- | --- | --- | --- | --- | --- |
| Iron-deficient diet | 0 days | 0 days | 2 days | 4 days | 8 days |
| RBCs (10^12^/L) | 10.40±0.81 | 10.88±0.30 | 10.83±0.30 | 10.68±0.22 | 10.5±0.48 |
| HGB (g/L) | 155.2±5.0 | 153.5±0.7 | 154.0±2.0 | 151.3±2.1 | 147.3±9.8 |
| HCT | 0.51±0.04 | 0.51±0.06 | 0.51±0.02 | 0.50±0.01 | 0.49±0.03 |
| MCV (fL) | 49.0±1.2 | 46.6±0.7 | 46.8±0.3 | 46.9±0.7 | 46.4±0.9 |
| MCH (pg) | 14.9±0.5 | 14.3±0.4 | 14.2±0.2 | 14.2±0.1 | 14.0±0.4 |
| MCHC (g/L) | 304.2±4.7 | 303.0±2.8 | 304.3±6.1 | 302.0±4.5 | 301.8±6.2 |

Two-month-old male *Fpn1^Alb/Alb;LysM/LysM^* mice were fed an AIN-76A (iron-deficient) diet (0.9 mg iron/kg) for 0, 2, 4, or 8 days (n=5 per group). Blood was then harvested for hematologic parameter analysis. Blood parameters of *Fpn1^flox/flox^* mice at day 0 were measured as a control. Results are presented as mean ± SD. *****P<0.05; ******P<0.01.
